# Maternal healthcare services use in Mwanza Region, Tanzania: a cross-sectional baseline survey

**DOI:** 10.1186/s12884-019-2653-4

**Published:** 2019-12-05

**Authors:** James Orwa, Michaela Mantel, Micheal Mugerwa, Sharon Brownie, Eunice Siaity Pallangyo, Loveluck Mwasha, Kahabi Isangula, Leonard Subi, Secilia Mrema, Grace Edwards, David Siso, Edna Selestine, Tumbwene Mwansisya, Columba Mbekenga, Marleen Temmerman

**Affiliations:** 1 Department of Population Health, Aga Khan University, Nairobi, Kenya; 2 Aga-Khan Health Services, Mwanza, Tanzania; 3 Research Associate, Green Templeton College, Oxford University, Oxford, UK; 4 Aga Khan University School of Nursing and Midwifery, Dar es Salaam, Tanzania; 5 Ministry of Health, Community Development, Gender, Elderly and Children, Dar es Salaam, Tanzania; 6 Regional Reproductive and Child Health Coordinator, Mwanza Region, Tanzania; 7 School of Nursing and Midwifery, Aga Khan University, Kampala, Uganda; 8 Aga Khan Foundation, Dar es Salaam, Tanzania

**Keywords:** Antenatal care, Health facility delivery, Postpartum care, Mwanza, Tanzania

## Abstract

**Background:**

Improving maternal health by reducing maternal mortality/morbidity relates to Goal 3 of the Sustainable Development Goals. Achieving this goal is supported by antenatal care (ANC), health facility delivery, and postpartum care. This study aimed to understand levels of use and correlates of uptake of maternal healthcare services among women of reproductive age (15–49 years) in Mwanza Region, Tanzania.

**Methods:**

A cross-sectional multi-stage sampling household survey was conducted to obtain data from 1476 households in six districts of Mwanza Region. Data for the 409 women who delivered in the 2 years before the survey were analyzed for three outcomes: four or more ANC visits (ANC4+), health facility delivery, and postpartum visits. Factors associated with the three outcomes were determined using generalized estimating equations to account for clustering at the district level while adjusting for all variables.

**Results:**

Of the 409 eligible women, 58.2% attended ANC4+, 76.8% delivered in a health facility, and 43.5% attended a postpartum clinic. Women from peri-urban, island, and rural regions were less likely to have completed ANC4+ or health facility delivery compared with urban women. Education and early first antenatal visit were associated with ANC4+ and health facility delivery. Mothers from peri-urban areas and those who with health facility delivery were more likely to attend postpartum check-ups.

**Conclusion:**

Use of ANC services in early pregnancy influences the number of ANC visits, leading to higher uptake of ANC4+ and health facility delivery. Postpartum check-ups for mothers and newborns are associated with health facility delivery. Encouraging early initiation of ANC visits may increase the uptake of maternal healthcare services.

## Background

Globally, there has been a decline of 45% per year since 1909 in maternal deaths and about 289,000 deaths estimated in 2013 [1]. Most of these deaths occur in limited resource settings and are preventable. In 2015, an estimated 303,000 women died following pregnancy and childbirth, with sub-Saharan Africa contributing more than half of these deaths [1, 2]. The Tanzania Demographic Health Survey (TDHS) 2015–2016 [3] reported Tanzania’s estimated maternal mortality ratio was 556 per 100,000 live births, reflecting an increase from 454 per 100,000 live births in the TDHS 2010 and the Population and Housing Census 2012 (432 per 100,000 live births). Although Tanzania has experienced a decline in early neonatal mortality from 40/1000 live births in 1999 to 25/1000 live births in 2015–2016, the rate remains higher than Sustainable Development Goal (SDG) targets [3]. This indicates that Tanzania is still far from meeting SDG targets of reducing maternal mortality to 70 per 100,000 live births and neonatal mortality to 12/1000 births by 2030 (SDG Targets 3.1 and 3.2, respectively) [4]. The main causes of maternal death among Tanzanian women are high rates of hemorrhage and eclampsia, which are detectable through attending antenatal care (ANC) services, along with indirect causes such as distance to health facilities [5, 6]. The availability and delivery of quality maternal healthcare throughout pregnancy, delivery, and the postpartum period are known to improve maternal health [7]. Maternal healthcare services (MHS), including ANC, health facility delivery, and postpartum check-ups, can play an important role in the reduction of maternal mortality and morbidity and remain pillars of safe motherhood [8].

According to the TDHS 2015–2016, 98% of Tanzanian women who delivered in the last 2 years had attended ANC at least once; however, only 51% attended the recommended four or more visits (ANC4+) [3]. The survey also showed that 63% of women delivered in health facilities and 64% were assisted by skilled birth attendants. However, uptake of recommended postpartum care in Tanzania is low, ranging from 25.8% in 2010 to 34% in 2015–2016; in addition, less than half (42%) of newborns were seen 2 days after delivery [3]. Mwanza Region had 96.6% ANC coverage, 53.3% health facilities deliveries, 54.2% skilled birth attendances, and 20.7% postpartum check-ups [3]. Other studies in Tanzania showed a 58.3% ANC4+ visits and 87.7% health facility delivery in Iringa district [9] and 56% health facilities deliveries in Biharamulo [10].

Several factors have been demonstrated to influence MHS attendance during the antenatal, delivery, and postpartum periods. These include factors such as higher parental education level, urban residence, richer household index, women’s level of autonomy in health expenditure, belonging to a minority ethnic group, and use of ANC during pregnancy (which is a significant factor for health facility delivery and postpartum check-ups) [11–14]. These factors are consistent with previous Tanzanian studies, which also found distance to the health facility and desire to avoid pregnancy influenced maternal healthcare attendance [15–18].

Although some studies on maternal health have been undertaken in Tanzania, few studies have investigated the uptake of MHS and associated determinants in the Mwanza Region. Available TDHS data have not been analyzed to identify the determinants of MHS use in Tanzania. Therefore, it is necessary to understand existing gaps in knowledge regarding factors that promote use of MHS to develop intervention tools that can support improved maternal outcomes. Understanding factors influencing use of MHS is crucial to inform future strategies to facilitate the development of policies to improve service use and thereby reduce maternal mortality; this will also help Tanzania to achieve the World Health Organization (WHO) recommendation of eight ANC contacts during pregnancy [19]. Therefore, this study aimed to determine the prevalence of ANC4+ visits, health facility deliveries, and postpartum care services use and their correlates among women of reproductive age using data from six districts in Mwanza Region.

## Methods

**Fig. 1:**
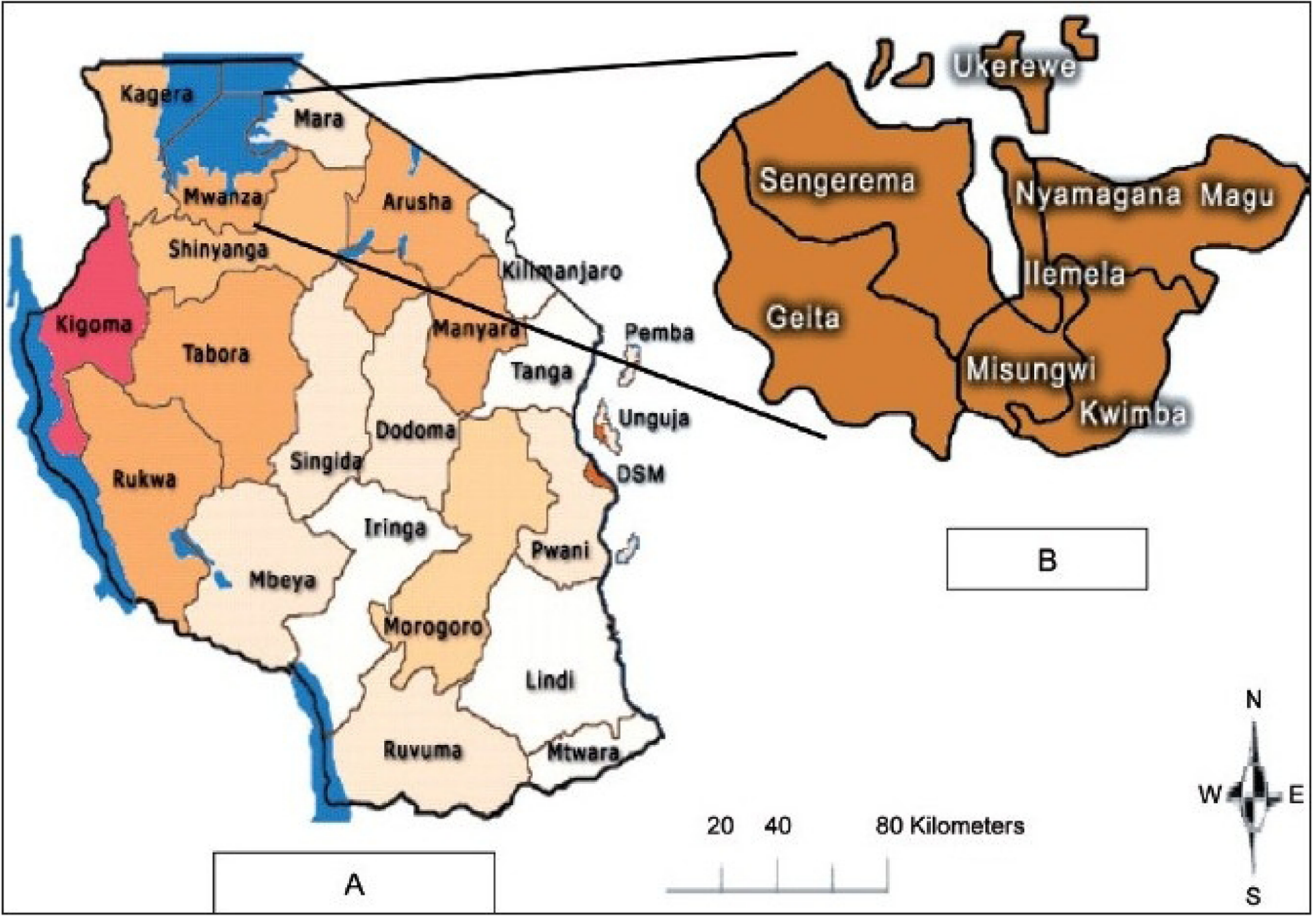
Map showing the districts of Mwanza Region, Tanzania [20]. Source: Mazigo HD, Okumu FO, Kweka EJ, Mnyone LL: Retrospective analysis of suspected rabies cases reported at bugando referral hospital, mwanza, Tanzania. *J Glob Infect Dis* 2010, 2(3):216–220

Data were collected as part of the 2017 baseline survey for the “Improving Access to Reproductive, Maternal and Newborn Health in Tanzania” (IMPACT) project. The IMPACT project aims to accelerate the reduction of maternal and newborn mortality by addressing major reproductive, maternal and newborn challenges in eight districts of Mwanza Region. This study was conducted in six of the eight districts: Ukerewe, Nyamagana, Illemela, Magu, Sengerema, and Buchosa districts excluding Kwimba and Misungwi districts that had an ongoing similar project (Fig. 1).

The present study used a descriptive, cross-sectional, multi-stage design to select eligible households for participation in the survey. With the help of village executive officers, a listing of all households was obtained for each district. This list was used as the master frame from which the number of households were randomly selected for the survey. In the first stage, 30 of 408 villages were selected across the six districts using probability proportional to the size of villages (number of households in the respective villages). The second stage involved random selection of households in each selected village. The required sample size for households was calculated to detect a change of 10% in skilled birth attendants between baseline and study endline, with a 95% level of significance, 0.05 margin of error, and 5.6 crude birth rate, using 2 as a design effect and a 10% non-response rate. This resulted in a required sample size of 1476 households, of which 1312 households’ members were present and consented.

No further sampling was undertaken in the sample households; all women in those households aged 15–49 years were eligible to participate. Women present in the household at the time of the visit that consented to participate were interviewed. Data were collected from August to September 2017. A total of 1612 women met the eligibility criteria but 1167 consented, resulting in a 72% response rate. Of the 1167 women who consented, 409 reported a live birth in the preceding 2 years.

### Ethical considerations

The baseline survey was approved by the National Institute for Medical Research in Tanzania (registration certificate: NIMR/HQR/R.8a/Vol.IX/2517) and the Institutional Review Board at Aga-Khan University in Dar-es-salaam, Tanzania. The Regional Medical Officer and district reproductive and child health coordinators authorized the survey. Village administrators granted permission to conduct the survey in households in their village of jurisdiction. All survey participants provided oral and written informed consent after receiving an explanation of the purpose of this study, duration of the interview, and their right to refuse or withdraw from the interviews at any time during the study process.

### Data collection

All data were collected through face-to-face interviews in either English or Swahili by the enumerators in a private place in the selected household (or in some cases in the homestead) using a translated electronic tool. Data collection was undertaken by teams of trained enumerators who were fluent in both English and Swahili. All enumerators participated in a 6-day training program covering data collection tools, interviewing skills, research ethics, and use of electronic devices (tablets) for data collection. The last day of the training was used for practical exercises in a nearby village, which was not part of the sample used for the baseline survey. Each district had a team of six enumerators. A team lead oversaw data collection and ensured that all data were uploaded onto the server. Data quality during and after the survey was ensured by setting validation checks in the electronic data collection forms, random spot checks on some households, and daily supervision of the data collection process. Any issues identified were discussed the following morning before the start of that day’s data collection.

### Measures

The selection of variables was based on a review of relevant literature. Three outcome measures of MHS use were evaluated: number of ANC visits, delivery in a health facility, and postpartum care. Based on the recommended standard of ANC4+ visits during pregnancy, a woman who attended at least four visits received a score of 1. Women that attended none or fewer than four visits received a score of 0. An ANC visit was defined as a woman that reported having visited a nurse, midwife, clinical officer, or medical doctor during their last pregnancy. Health facility delivery was assessed by a question about the place of delivery of the last pregnancy, and was coded dichotomously as 1 if a woman delivered in a formal private/public medical facility with the help of a health professional, or 0 if she delivered at home or on the way to a medical facility. Postpartum care was assessed by asking each woman if a healthcare worker had checked on her health after delivery regardless of the place of delivery. Postpartum care was coded a 1 if a woman received any form of postpartum care (irrespective of number of days after delivery and place of delivery) and 0 if she did not receive a postpartum check-up.

Explanatory variables included: district of residence, maternal age at time of the survey, marital status, and gestational age at first ANC visit, wealth quintile, maternal education, and whether the last pregnancy was wanted. Age was recorded on a continuous scale and later categorized into four groups (15–19, 20–29, 30–39, and 40–49 years). Marital status was collected as a categorical variable (currently married, in-union, and not in-union). The in-union group was merged with the currently married group and coded as 1; the not in-union group was coded as 0. Gestational age at first ANC visit was collected as a continuous variable in weeks and then categorized as first, second, and third trimester/no ANC visit. This was then dichotomized as a first trimester group and a second/third trimester and no ANC group. This allowed comparison of women who had ANC during their first trimester and those who did not attend/had late ANC visits. Wealth index (generated through principal component analysis based on household assets) was grouped into quintiles: poorest, poor, middle, rich, and richest. Maternal education level was dichotomized as primary education or below and secondary education or above. Assistance during delivery was categorized as skilled delivery if the woman was assisted by a nurse, clinical officer, or medical doctor. Otherwise, the delivery was categorized as unskilled. Finally, women that reported that their last pregnancy was wanted were coded as 1, and pregnancies that were not wanted (e.g., mistimed) were coded as 0.

The community level variable identifying district and village of residence was only used in the descriptive table and was not included in the analysis because there was no variation in outcomes at this level as determined by the intra-class correlation coefficient; therefore, it was included in the model as a fixed effect. District of residence was coded to compare urban, rural, peri-urban, and island locations. Magu and Sengerema were classified as peri-urban districts, Ukerewe as an island district, Nyamagana and Illemela as urban districts, and Buchosa as a rural district.

### Data analysis

Data were analyzed in three steps. First, data were explored descriptively using frequency (percentages) and median (interquartile range [IQR]). Second, bivariate analyses were completed using chi-square tests to examine associations between individual factors and different outcome measures. This was also used to select variables to be retained in the multivariate analysis. Third, multivariate generalized estimation equation (GEE) regression analysis which is a form of logistic regression for clustered data was conducted to determine the adjusted effects of all explanatory variables on the three outcome variables. Three models were examined to assess the adjusted effects of predictors on ANC4+, facility delivery, and postpartum care. Variables that had a *p*-value < 0.25 in the bivariate analysis were included in the multivariate models. Stata version 12 (College station, Texas, USA) was used for all analyses. Data were de-identified by deleting all personal identifiers before analysis to ensure participants’ anonymity and confidentiality throughout the study.

## Results

### Sample characteristics

In total, 409 women gave birth in the 2 years before this survey, of which the majority were from Buchosa district (*n* = 144; 35.2%). The median age (IQR) was 27 (23–33) years. There were 34 (8.3%) births among adolescents, with a median age of 18 years. The majority of women were married/in-union (*n* = 329, 80.4%) and had primary education or below. Just over half of the women (*n* = 216, 52.8%) reported not wanting their last pregnancy at that time. Over 96% (*n* = 395) of the women reported at least one ANC visit during their last pregnancy, but only 234 (57.2%) reported ANC4+ visits. Among those who had at least one ANC visit, 95 (23.2%) attended their first ANC visit in the first trimester (12 weeks gestation), and over three-quarters of the women (*n* = 314, 76.8%) gave birth in a health facility. Skilled health providers assisted three in every four facility deliveries (308, 75.3%). Finally, 43.5% (178) of women received a postpartum health check-up (Table 1).

**Table 1:** Sociodemographic characteristics of participating women (*N* = 409)

Characteristics	Frequency (%)
District of residence	
Illemela (U)	85 (20.8)
Nyamagana (U)	68 (16.6)
Magu (P-U)	57 (13.9)
Sengerema (P-U)	34 (8.3)
Buchosa (R)	144 (35.2)
Ukerewe (I)	21 (5.1)
Maternal age, years,	
Median (IQR)	27 (23–33)
15–19	34 (8.3)
20–29	214 (52.3)
30–39	134 (32.8)
40–49	27 (6.6)
Marital status	
Currently married	329 (80.4)
In-union	23 (5.6)
Not in-union	57 (13.9)
Level of education	
Primary and below	329 (80.4)
Secondary	73 (17.9)
Higher	7 (1.7)
Wealth quintiles	
Poorest	94 (23.0)
Poor	116 (28.4)
Middle	62 (15.2)
Rich	89 (21.8)
Richest	48 (11.7)
Pregnancy wanted at that time	
Yes	193 (47.2)
No	216 (52.8)
Place of delivery	
Home	76 (18.6)
On the way	10 (2.4)
Health facility	314 (76.8)
Other places	9 (2.2)
ANC attendance	
None	14 (3.4)
1–3 visits	161 (39.4)
4+ visits	234 (57.2)
Timing of initial ANC	
First trimester	95 (23.2)
Second trimester	265 (64.8)
Third trimester	42 (10.3)
No ANC visit	7 (1.7)
Assisted delivery	
Skilled delivery^a^	308 (75.3)
Unskilled delivery	101 (24.7)
Any postpartum check-up	
Yes	178 (43.5)
No	231 (56.5)

^a^delivery conducted by nurse/midwife, medical officer, clinical officer

*R* rural, *P-U* peri-urban, *U* urban, *I* island

### Bivariate analysis of maternal health service utilization

There was a significant association between district of residence and ANC4+ attendance. Marital status was only associated with postpartum check-up, with a higher proportion of not in-union women (*n* = 34, 59.6%) receiving this service compared with married/in-union women (*n* = 144; 40.9%). Women with a secondary education and above were significantly more likely to have had ANC4+ visits (*n* = 59, 73.8%) and health facility delivery (*n* = 75, 92.5%) compared with women with primary education or below, but there was no difference in the likelihood of receiving a postpartum check-up. The wealth index was significant in predicting ANC4+ visits, with those in the lowest and highest wealth categories being most likely to seek care. Women who had attended ANC in their first trimester were significantly more likely to have ANC4+ visits (*n* = 74, 77.9%) and to deliver in a health facility (*n* = 34, 59.6%) than other women; however, timing of initiation of ANC did not predict the likelihood of postpartum care. Women who wanted to be pregnant at the time of their last pregnancy were more likely to deliver in a healthcare facility, but this was not significantly associated with the likelihood of ANC4+ or a postpartum check-up. Finally, women who attended ANC4+ visits (*n* = 188; 59.9%) were likely to deliver in health facility and receive postpartum check-ups (*n* = 152; 48.4%) than women who delivered outside of a health facility (Table 2).

**Table 2:** Maternal health service utilization according to sample characteristics

Background characteristics	Total (*N* = 409)	Antenatal care (4+) (*n* = 234)		Health facility delivery (*n* = 314)		Postpartum check-up (*n* = 178)	
		n (%)	*p*-value	n (%)	*p*-value	n (%)	*p*-value
District of residence			< 0.0001		< 0.0001		0.005
Urban	165	115 (69.7)		149 (90.3)		71 (43.0)	
Peri-urban	142	73 (51.4)		105 (73.9)		76 (53.5)	
Rural	68	33 (48.5)		48 (70.6)		20 (29.4)	
Island	34	13 (38.2)		12 (35.3)		11 (32.4)	
Age group, years			0.487		0.351		0.195
15–19	34	18 (52.9)		30 (88.2)		16 (47.1)	
20–29	214	124 (57.9)		164 (76.6)		83 (38.8)	
30–39	134	80 (59.7)		101 (75.4)		64 (47.8)	
40–49	27	12 (44.4)		19 (70.4)		15 (55.6)	
Marital status			0.297		0.935		0.008
Married/in-union	352	205 (58.2)		270 (76.7)		144 (40.9)	
Not in-union	57	29 (50.9)		44 (77.2)		34 (59.6)	
Level of education			0.001		< 0.0001		0.583
Primary and below	329	175 (53.2)		239 (72.6)		141 (42.9)	
Secondary and above	80	59 (73.8)		75 (93.8)		37 (46.3)	
Wealth quintile			0.033		0.361		0.116
Poorest	94	61 (64.9)		71 (75.5)		51 (54.3)	
Poor	116	61 (52.6)		95 (81.9)		50 (43.1)	
Middle	62	32 (51.6)		46 (74.2)		25 (40.3)	
Rich	89	45 (50.6)		63 (70.8)		31 (34.8)	
Richest	48	35 (72.9)		39 (81.3)		21 (43.8)	
ANC initial visit, trimester			< 0.0001		0.007		0.388
First	95	74 (77.9)		83 (87.4)		41 (43.2)	
Second	265	159 (60.0)		199 (75.1)		120 (45.3)	
Third/None	49	1 (2.0)		32 (65.3)		17 (34.7)	
Pregnancy wanted at that time			0.474		0.021		0.424
Yes	193	114 (59.1)		158 (81.9)		88 (45.6)	
No	216	120 (55.6)		156 (72.2)		90 (41.7)	
Place of delivery			0.048				< 0.0001
Home/on the way	95	46 (48.4)		n/a		26 (27.4)	
Health sector	314	188 (59.9)		n/a		152 (48.4)	

*ANC* antenatal care, *n/a* not applicable

### Multivariable analysis of MHS use

The multivariable analysis is shown in Table 3, it showed that only women with an island district of residence were significantly less likely to have ANC4+ visits than those from urban districts (odds ratio [OR] 0.33, 95% confidence interval [CI]: 0.12–0.90) (Table 3). Women with a secondary education and above were twice as likely to attend ANC4+ visits (OR 1.85, 95% CI: 0.96–3.55) compared with women with a primary education and below; however, the association was not significant after adjusting for other factors. Women who initiated ANC visits in the second/third trimester were significantly less likely to complete ANC4+ visits than women who attended ANC in the first trimester. Other variables did not show significant associations with ANC4+ visits after adjusting for confounders.

**Table 3:** Adjusted analysis of maternal health service use outcomes by sociodemographic factors

Background characteristics	Model I			Model II			Model III		
	Antenatal care			Delivery-health sector			Post-natal care-mother		
	OR	95% CI	*p*-value	OR	95% CI	*p*-value	OR	95% CI	*p*-value
District of residence									
Urban	Ref			Ref			Ref		
Peri-urban	0.61	0.33–1.13	0.119	0.32	0.19–0.56	< 0.0001	2.08	1.25–3.48	0.005
Rural	0.55	0.26–1.16	0.116	0.23	0.12–0.41	< 0.0001	0.65	0.34–1.24	0.189
Island	0.33	0.12–0.90	0.031	0.05	0.03–0.11	< 0.0001	1.35	0.58–3.17	0.485
Maternal age, years	1.01	0.98–1.05	0.433	1.01	0.97–1.04	0.683	1.02	0.99–1.06	0.154
Marital status									
Not in-union	Ref			Ref			Ref		
Married/in-union	1.32	0.68–2.57	0.415	0.97	0.46–2.06	0.941	0.39	0.21–0.73	0.003
Level of education									
Primary and below	Ref			Ref			Ref		
Secondary and above	1.85	0.96–3.55	0.065	4.51	1.61–12.5	0.004	0.93	0.53–1.62	0.790
Wealth quintile									
Poorest	1.39	0.64–3.02	0.400	0.81	0.36–1.80	0.601	2.45	1.19–5.06	0.015
Poor	0.77	0.38–1.58	0.483	1.76	0.78–3.94	0.171	1.31	0.66–2.60	0.436
Middle	Ref			Ref			Ref		
Rich	0.73	0.34–1.54	0.406	0.75	0.33–1.65	0.468	0.96	0.47–1.99	0.919
Richest	1.20	0.45–3.21	0.717	0.41	0.14–1.17	0.097	1.53	0.64–3.68	0.337
Pregnancy wanted at that time									
Yes	Ref								
No	1.28	0.80–2.05	0.295	0.65	0.39–1.11	0.116	0.87	0.56–1.34	0.538
ANC initial visit, trimester	Ref								
First	Ref								
Second	0.43	0.24–0.76	0.003	0.44	0.21–0.91	0.026	1.19	0.72–1.97	0.504
Third/None	0.01	0.0008–0.047	< 0.0001	0.49	0.19–1.25	0.134	0.72	0.33–1.57	0.410
Place of delivery									
Home/on the way	Ref						Ref		
Health facility	0.94	0.52–1.68	0.827				2.87	1.61–5.12	< 0.0001

*Ref* reference categories, *ANC* antenatal care, *OR* odds ratio

Women living in peri-urban, rural, and island districts were significantly less likely to deliver in a health facility than women living in urban districts (OR 0.32, 95% CI: 0.19–0.57; OR 0.23, 95% CI: 0.12–0.42; and OR 0.05, 95% CI: 0.03–0.12, respectively). Women with a secondary education or above were over four times more likely to deliver at a health facility compared with those with a primary education or below (OR 4.51, 95% CI: 1.61–12.50). Finally, women who attended their first ANC visit during the second trimester were less likely to deliver in a health facility than women who attended their first ANC visit during the first trimester (OR 0.44, 95% CI: 0.21–0.91).

With respect to postpartum care, women from peri-urban districts were twice as likely to receive postpartum check-ups as women from urban districts (OR 2.08, 95% CI: 1.25–3.48). Women who were married/in-union were less likely to receive postpartum check-ups (OR 0.39, 95% CI: 0.99–0.73) than the not in-union group. Maternal age, level of education, whether the pregnancy was wanted, and time of initiation of ANC visits were not significant in terms of receiving postpartum care. However, women from households with the poorest wealth quintile were 2.45 times more likely to receive postpartum care compared with women from the middle wealth quintile (OR 2.45, 95% CI: 1.19–5.06). Finally, women who delivered in a health facility were almost three times more likely to receive a postpartum check-up compared with women who did not deliver in a health facility (OR 2.87, 95% CI: 1.61–5.12).

## Discussion

This study examined three components of MHS and their correlates among women of reproductive age (15–49 years) in Mwanza Region, Tanzania. The findings showed that the percentages of women who attended ANC4+ visits and delivered in health facility were higher than the findings from the TDHS 2015–2016 for Mwanza Region [3]. The proportion of health facility deliveries was comparable with that in Mbarali District, Tanzania, where 70% of women delivered in a health facility [16]. The analysis of these results indicated that the efforts of the Government of Tanzania and other partners in improving access to MHS services appeared to positively impact the coverage of ANC4+ attendance and health facility deliveries.

This study found significant differences in ANC4+ attendance and health facility delivery by area of residence, with women from urban districts showing higher use of ANC services compared with women outside of urban districts. These findings were consistent with recent TDHS results and a Nigerian study [21]. This suggested that area of residence may play a role in ANC visits and facility delivery, with those living further away from urban centers being less likely to receive/use care. A reason for this variation may be that women in rural areas may still have overriding cultural and social norms guiding their care-seeking behavior, such as strong faith in traditional birth attendants or desire to bury the placenta near their home. These factors may have negative influences on MHS use, compared with urban women who may be more educated and less influenced by traditional beliefs [22, 23]. The low use of MHS outside urban areas may also be attributable to health system factors such as scarcity of health facilities, poor infrastructure, and lack (or absence) of healthcare providers in low volume facilities, especially in rural and island districts [24], as well as abusive and disrespectful maternity care [25]. Identification of factors that may explain these differences is important as Tanzania works to achieve higher rates of MHS use.

Our study found significantly higher rates of health facility delivery among women with a secondary school education and above compared with women with less education. This finding was consistent with previous studies that showed a significant link between higher levels of maternal education and higher levels of maternal healthcare use [11, 26–28] and contrasted with the findings of an Ethiopian study [29]. Educated women are believed to be more willing to seek healthcare because they have higher levels of knowledge through health education on the benefits of ANC and health facility delivery services. Most of these women also have greater employment opportunities in urban areas and therefore may be more likely to live in urban areas where there are more health facilities. However, even after adjusting for district, education level was still significant for MHS use. This indicated that access to education may also give women autonomy and capacity to make their own decisions regarding health service use [30].

The present findings regarding the impact of wealth and income on MHS use were equivocal. In the bivariate analysis, wealth was significant but the relationship was U-shaped, indicating that those in the lowest and highest categories had an advantage in MHS use compared with those in the middle wealth categories. However, after adjusting for other factors (e.g., geographic district and maternal education), there was no significant association between wealth quintile and use of ANC4+ or health facility delivery services. The only significant finding was that women in the lowest wealth category were more likely to receive postpartum care compared with those in the middle wealth categories. These findings were consistent with a study on determinants of postnatal care in rural Tanzania [16], but contrasted with findings of other studies that reported significant associations between wealth index and MHS use [27, 29, 31, 32]. This suggested that offering ANC services free of charge may not be sufficient to improve the use of these services, as other factors (e.g., cost of transportation to the health facility and finding money for other treatment services) may be impediments to accessibility and use of these services among those in lower income categories. In addition, maternal education and geographic proximity appeared to be more important in predicting use of services for delivery, whereas initiating earlier ANC visits appeared to be more important for ANC4+ visits. In fact, timing of initiation of ANC services appeared to be the strongest predictor of ANC4+ and was also a significant predictor of health facility delivery. Women who started their ANC visits during the second trimester had increased odds of attending ANC4+ and health facility delivery. These findings are supported by previous studies [28, 33, 34] as well as by the recent TDHS [3], which found that women who attended ANC4+ were more likely to deliver in a health facility and receive postpartum check-ups than women who did not attend ANC4+. These results suggest that ANC services during the early weeks of pregnancy allow women time to attend all scheduled ANC visits and receive education on the health benefits of hospital delivery. However, geography may be an overriding factor, as a study in rural Tanzania did not find an association between the number of ANC visits and health facility delivery [35]. Moreover, the present study found the geographic factor was the strongest predictor of location of delivery, with those further away from urban locations being less likely to deliver in a facility.

Finally, there was a significant association between delivery location and use of postpartum services, with women who delivered in a health facility being more likely to receive postpartum check-ups compared with those who did not deliver in a health facility. This contrasted with a study from Ethiopia that found an association between health facility delivery and postpartum care (although with reduced likelihood) [36]. Women who delivered in health facility were likely to be assisted by skilled health professionals who follow standard procedures of providing postnatal care to the mother and newborn within 48 h after delivery, as per the Tanzania national guideline. Moreover, missing this service may put the life of the mother and the infant at risk [37]. It may be possible that some women missed postpartum check-ups because of inadequate staffing and lack of equipment and supplies in health centers and dispensaries, as most health facilities have only one nurse to attend to all patients [38]. This staffing problem coupled with dismissive or disrespectful treatment of women by health workers in Tanzania has been highlighted elsewhere, with studies showing that this results in low use of MHS [25, 33, 39, 40]. The positive association between health facility delivery and postpartum check-ups may be attributable to the fact that women who gave birth in a health facility had a greater chance of receiving health education related to postpartum check-ups at the time of delivery, and therefore gained knowledge on the types, benefits, and availability of postpartum services during their stay in the health institution [30].

### Limitations

Although this study contributes to the growing literature examining MHS use, there are some limitations that need to be mentioned. First, this study was undertaken among women of reproductive age who had given birth in the last 2 years. This time lapse may have created recall bias, especially about the number of ANC visits during the last pregnancy. However, the survey tools were designed with added probes questions to help the participants remember the responses. Second, the number of adolescents included in the survey was not likely to be representative of the adolescent population in the Mwanza Region. Cultural issues (e.g., sex before marriage), government policy (e.g., denying continuation of school education after delivery) and stigma from parents and adults in the community (e.g., that the girls had put themselves at risk for pregnancy) around adolescent pregnancy may have hindered the participation of most teenagers. Most adolescents were also in school at time of the interview, and few who were married or had dropped out of school were found at home during the study visits. Third, some variables that might impact the study findings (e.g., religion, distance to the closest health facility, parity, and other cultural factors) were not analyzed as they were not collected in the dataset used. Finally, the study adopted a cross-sectional design, which limit inferring causation and has a possibility of reverse-causality.

## Conclusion

This study showed that women with primary school or below education as well as those living in rural or island districts were less likely to use MHS. Women with secondary education or above showed higher use of these services than less educated women. This indicates disproportionate use of MHS across education levels, despite the high demand for these services reported in research in southern Tanzania [41] that identified regional disparity in access to maternal healthcare. This survey also found ANC care was significantly linked to health facility delivery, which in turn was significantly associated with postpartum service use. The results call for community-based interventions to create increased awareness and early initiation of ANC services, especially among women with no/low education and women in rural and remote (island) areas. Efforts should be made to strengthen ANC4+ attendance, with the expectation that this will lead to higher use of health facility delivery services. This is particularly relevant given that Tanzania envisages adopting the WHO recommendations for eight ANC contacts, as this has been shown to contribute to increased use of delivery services and increased uptake of postpartum services. In remote areas with limited access to health facilities, regional and district health planning and programming should consider alternative and innovative strategies and interventions to ensure remote communities have access to ANC, delivery, and postpartum services. For example, this could include innovations such as provision of mobile health services or temporary shelters for MHS. These initiatives may be considered for further evaluation in light of feasibility, acceptability and cost-effectiveness.

Finally, apart from the policy of free ANC and health facility delivery in Tanzania, other significant factors such as demographic, societal, and cultural factors should be considered in further studies as they impact women’s health and use of MHS. Specifically, as this was a quantitative survey and responses were restricted to options listed in the questionnaire, it is recommended that further qualitative research is conducted to understand and verify the present findings in relation to the sociocultural and economic context of the studied communities. To our knowledge, this is the first survey to explore MHS use at the household level in six districts of Mwanza Region. The results of the survey may provide evidence and guidance for policy- and decision-makers and project planers and implementers to increase the coverage and quality of key MHS, thereby increasing access and ultimately contributing to reduced maternal mortality.

## Data Availability

The data that support the findings of this study are available from Aga Khan University Monitoring and Evaluation Research Unit (MERL) but restrictions apply to the availability of these data, which were used under license for the current study, and so are not publicly available. Data are however available from the authors upon reasonable request and with permission of Aga Khan University Monitoring and Evaluation Research Unit (MERL).

## Abbreviations

ANC: Antenatal care
IMPACT: Improving access to reproductive, maternal and newborn health in Tanzania
MHS: Maternal health services
MHS: Maternal health-care services
NIMRI: National institute for medical research
SDG: Sustainable development goal
TDHS: Tanzania demographic health survey
WHO: World health organization

## References

[1] Tessema GA, Laurence CO, Melaku YA, Misganaw A, Woldie SA, Hiruye A, Amare AT, Lakew Y, Zeleke BM, Deribew A (2017). Trends and causes of maternal mortality in Ethiopia during 1990-2013: findings from the global burden of diseases study 2013. BMC Public Health.

[2] Alkema L, Chou D, Hogan D, Zhang S, Moller A-B, Gemmill A, Fat DM, Boerma T, Temmerman M, Mathers C (2016). Global, regional, and national levels and trends in maternal mortality between 1990 and 2015, with scenario-based projections to 2030: a systematic analysis by the UN maternal mortality estimation inter-agency group. Lancet.

[3] Statistics TBo (2016). Tanzania demographic and health survey 2016. National Bureau of Statistics Dar es Salaam, Tanzania.

[4] World Health Organization. World health statistics - monitoring health for the SDGs. World Health Organization; 2016. 1.121.

[5] Illah E, Mbaruku G, Masanja H, Kahn K (2013). Causes and risk factors for maternal mortality in rural Tanzania--case of Rufiji health and demographic surveillance site (HDSS). Afr J Reprod Health.

[6] Hanson C, Cox J, Mbaruku G, Manzi F, Gabrysch S, Schellenberg D, Tanner M, Ronsmans C, Schellenberg J (2015). Maternal mortality and distance to facility-based obstetric care in rural southern Tanzania: a secondary analysis of cross-sectional census data in 226 000 households. Lancet Glob Health.

[7] Tunc Ӧ, Were W, Maclennan C, Oladapo O, Bahl R, Daelmans B, Mathai M, Say L, Kristensen F, Temmerman M (2015). Quality of care for pregnant women and newborns—the WHO vision. BJOG.

[8] Abel Ntambue ML, Francoise Malonga K, Dramaix-Wilmet M, Donnen P (2012). Determinants of maternal health services utilization in urban settings of the Democratic Republic of Congo--a case study of Lubumbashi City. BMC Pregnancy Childbirth.

[9] Straneo M, Fogliati P, Pellis I, Goodman C, Riva DD, Kisika F, Mpuya E, Putoto G (2016). On the way to universal coverage of maternal services in Iringa rural district in Tanzania. Who is yet to be reached?. Afr Health Sci.

[10] Mageda K, Mmbaga EJ (2015). Prevalence and predictors of institutional delivery among pregnant mothers in Biharamulo district, Tanzania: a cross-sectional study. Pan Afr Med J.

[11] Tarekegn SM, Lieberman LS, Giedraitis V (2014). Determinants of maternal health service utilization in Ethiopia: analysis of the 2011 Ethiopian demographic and health survey. BMC Pregnancy Childbirth.

[12] Rutaremwa G, Wandera SO, Jhamba T, Akiror E, Kiconco A (2015). Determinants of maternal health services utilization in Uganda. BMC Health Serv Res.

[13] Birmeta K, Dibaba Y, Woldeyohannes D (2013). Determinants of maternal health care utilization in Holeta town, Central Ethiopia. BMC Health Serv Res.

[14] Abor PA, Abekah-Nkrumah G, Sakyi K, Adjasi CKD, Abor J. The socio-economic determinants of maternal health care utilization in Ghana. Int J Soc Econ. 2011;38:628–48.

[15] Gupta S, Yamada G, Mpembeni R, Frumence G, Callaghan-Koru JA, Stevenson R, Brandes N, Baqui AH (2014). Factors associated with four or more antenatal care visits and its decline among pregnant women in Tanzania between 1999 and 2010. PLoS One.

[16] Mohan D, Gupta S, LeFevre A, Bazant E, Killewo J, Baqui AH (2015). Determinants of postnatal care use at health facilities in rural Tanzania: multilevel analysis of a household survey. BMC Pregnancy Childbirth.

[17] Ng’anjo Phiri S, Kiserud T, Kvale G, Byskov J, Evjen-Olsen B, Michelo C, Echoka E, Fylkesnes K (2014). Factors associated with health facility childbirth in districts of Kenya, Tanzania and Zambia: a population based survey. BMC Pregnancy Childbirth.

[18] Lwelamira J, Safari J, Stephen A (2015). Utilization of maternal postnatal care services among women in selected villages of Bahi District, Tanzania. Curr Res J Soc Sci.

[19] WHO. WHO | New guidelines on antenatal care for a positive pregnancy experience: WHO; 2016. 28079998

[20] Mazigo HD, Okumu FO, Kweka EJ, Mnyone LL (2010). Retrospective analysis of suspected rabies cases reported at bugando referral hospital, Mwanza, Tanzania. J Glob Infect Dis.

[21] Fagbamigbe AF, Idemudia ES (2017). Wealth and antenatal care utilization in Nigeria: policy implications. Health Care Women Int.

[22] Adewuyi EO, Auta A, Khanal V, Bamidele OD, Akuoko CP, Adefemi K, Tapshak SJ, Zhao Y (2018). Prevalence and factors associated with underutilization of antenatal care services in Nigeria: A comparative study of rural and urban residences based on the 2013 Nigeria demographic and health survey. PLoS One.

[23] Yasuoka J, Nanishi K, Kikuchi K, Suzuki S, Ly P, Thavrin B, Omatsu T, Mizutani T (2018). Barriers for pregnant women living in rural, agricultural villages to accessing antenatal care in Cambodia: a community-based cross-sectional study combined with a geographic information system. PLoS One.

[24] Kruk ME, Hermosilla S, Larson E, Vail D, Chen Q, Mazuguni F, Byalugaba B, Mbaruku G (2015). Who is left behind on the road to universal facility delivery? A cross-sectional multilevel analysis in rural Tanzania. Tropical Medicine and International Health.

[25] Kruk ME, Kujawski S, Mbaruku G, Ramsey K, Moyo W, Freedman LP (2018). Disrespectful and abusive treatment during facility delivery in Tanzania: a facility and community survey. Health Policy Plan.

[26] Rashid M, Antai D, Antai D (2014). Socioeconomic position as a determinant of maternal healthcare utilization: a population-based study in Namibia. J Res Health Sci.

[27] Ochako R, Gichuhi W (2016). Pregnancy wantedness, frequency and timing of antenatal care visit among women of childbearing age in Kenya. Reprod Health.

[28] Dahiru T, Oche OM (2015). Determinants of antenatal care, institutional delivery and postnatal care services utilization in Nigeria. Pan Afr Med J.

[29] Wilunda C, Quaglio G, Putoto G, Takahashi R, Calia F, Abebe D, Manenti F, Dalla Riva D, Betrán AP, Atzori A (2015). Determinants of utilisation of antenatal care and skilled birth attendant at delivery in south west Shoa zone, Ethiopia: A cross sectional study. Reprod Health.

[30] Gebeyehu Workineh Y (2014). Factors affecting utilization of postnatal Care Service in Amhara Region, Jabitena District, Ethiopia. Sci J Public Health.

[31] Tsawe M, Moto A, Netshivhera T, Ralesego L, Nyathi C, Susuman AS (2015). Factors influencing the use of maternal healthcare services and childhood immunization in Swaziland. Int J Equity Health.

[32] Exavery A, Kanté AM, Njozi M, Tani K, Doctor HV, Hingora A, Phillips JF (2014). Access to institutional delivery care and reasons for home delivery in three districts of Tanzania. Int J Equity Health.

[33] Kujawski S, Mbaruku G, Freedman LP, Ramsey K, Moyo W, Kruk ME (2015). Association Between Disrespect and Abuse During Childbirth and Women’s Confidence in Health Facilities in Tanzania. Matern Child Health J.

[34] Darega B, Dida N, Tafese F, Ololo S (2016). Institutional delivery and postnatal care services utilizations in Abuna Gindeberet District, west Shewa, Oromiya region, Central Ethiopia: a community-based cross sectional study. BMC Pregnancy Childbirth.

[35] Choe SA, Kim J, Kim S, Park Y, Kullaya SM, Kim CY (2016). Do antenatal care visits always contribute to facility-based delivery in Tanzania? A study of repeated cross-sectional data. Health Policy Plan.

[36] Angore BN, Tufa EG, Bisetegen FS (2018). Determinants of postnatal care utilization in urban community among women in Debre Birhan Town, Northern Shewa, Ethiopia. J Health Popul Nutr.

[37] Khanal V, Adhikari M, Karkee R, Gavidia T (2014). Factors associated with the utilisation of postnatal care services among the mothers of Nepal: Analysis of Nepal Demographic and Health Survey 2011. BMC Womens Health.

[38] Mrisho M, Obrist B, Schellenberg JA, Haws RA, Mushi AK, Mshinda H, Tanner M, Schellenberg D (2009). The use of antenatal and postnatal care: perspectives and experiences of women and health care providers in rural southern Tanzania. BMC Pregnancy Childbirth.

[39] Manzi F, Schellenberg JA, Hutton G, Wyss K, Mbuya C, Shirima K, Mshinda H, Tanner M, Schellenberg D (2012). Human resources for health care delivery in Tanzania: a multifaceted problem. Hum Resour Health.

[40] Sando D, Ratcliffe H, McDonald K, Spiegelman D, Lyatuu G, Mwanyika-Sando M, Emil F, Wegner MN, Langer A (2016). The prevalence of disrespect and abuse during facility-based childbirth in urban Tanzania. BMC Pregnancy Childbirth.

[41] Hanson C, Gabrysch S, Mbaruku G, Cox J, Mkumbo E, Manzi F, Schellenberg J, Ronsmans C (2017). Access to maternal health services: geographical inequalities, United Republic of Tanzania. Bull World Health Organ.

